# Anti-obesity effect of intranasal administration of galanin-like peptide (GALP) in obese mice

**DOI:** 10.1038/srep28200

**Published:** 2016-06-21

**Authors:** Haruaki Kageyama, Kanako Shiba, Satoshi Hirako, Nobuhiro Wada, Satoru Yamanaka, Yukinori Nogi, Fumiko Takenoya, Naoko Nonaka, Tsutomu Hirano, Shuji Inoue, Seiji Shioda

**Affiliations:** 1 Division of Nutrition, Faculty of Health Care, Kiryu University, Gunma 379-2392, Japan; 2 Department of Anatomy, Showa University School of Medicine, Tokyo, 142-8555, Japan; 3 Department of Health and Nutrition, University of Human Arts and Sciences, Saitama 339-8539, Japan; 4 Department of Internal Medicine, Graduate school of Medicine, University of Tokyo, Tokyo 113-8655, Japan; 5 Department of Medicine, Division of Diabetes, Metabolism, and Endocrinology, Showa University School of Medicine, Tokyo 142-8555, Japan; 6 Department of Exercise and Sports Physiology, Hoshi University School of Pharmacy and Pharmaceutical Science, Tokyo 142-8501, Japan; 7 Department of Oral Anatomy and Developmental Biology, School of Dentistry, Showa University, Tokyo 142-8555, Japan; 8 Hoshi University School of Pharmacy and Pharmaceutical Sciences, Global Research Center for Innovative Life Science, Peptide Drug Innovation, Tokyo 142-8501, Japan

## Abstract

Galanin-like peptide (GALP) has an anti-obesity effect in rats and mice. It has been reported that the uptake of GALP by the brain is higher after intranasal administration than with intravenous injection. This study therefore aimed to clarify the effect of intranasal administration of GALP on the feeding behavior of lean and obese mice. Autoradiography revealed the presence of ^125^I-GALP in the olfactory bulb and the brain microcirculation. The body weights of *ob/ob* mice gradually increased during vehicle treatment, but remained unchanged in response to repeated intranasal administration of GALP, with both *ob/ob* and diet-induced obese mice displaying significantly decreased food intake, water intake and locomotor activity when treated with GALP. These results suggest that intranasal administration is an effective route whereby GALP can exert its effect as an anti-obesity drug.

Galanin-like peptide (GALP) is a 60 amino acid neuropeptide that was originally isolated from porcine hypothalamus using a binding assay for galanin receptors (GALRs), which belong to the G protein-coupled receptor family^1^. The 9–21 amino acid sequence of GALP is identical to that of the first 13 amino acids of galanin. There are three subtypes of GALRs: GALR1, GALR2 and GALR3. Although GALP reportedly binds GALR, it reduces food intake and body weight in both GALR1 and GALR2 knockout mice, similar to the situation in wild-type mice^2^. It is therefore possible that it is GALR3 that mediates feeding behavior. However, it has been reported that central administration of a GALR2/3 agonist has no effect on food intake, body weight or body temperature in rodents^3^. These results suggest that GALP and galanin act through different receptor-mediated pathways to exert their effects on the regulation of feeding. In other words, it is possible that GALP mediates its effect via a yet-to-be-identified GALP receptor.

While GALP-producing neurons are present in the arcuate nucleus of the hypothalamus in the rat, mouse and macaque^4^,^5^,^6^, their distribution in the human brain is unknown. More than 85% of GALP-containing neurons express the leptin receptor^7^, with intracerebroventricular infusion of leptin elevating the number of GALP mRNA-expressing neurons. In contrast, the expression of GALP mRNA in the brain is markedly reduced in leptin-deficient *ob/ob* mice, leptin receptor-deficient *db/db* mice and Zucker fatty rats, compared with wild-type animals^5^. However, intracerebroventricular infusion of leptin in *ob/ob* mice restores both the number of GALP mRNA-expressing neurons and their GALP mRNA content^5^. These observations demonstrate that GALP mRNA expression is induced by leptin through a direct action on the brain.

In rats, the intracerebroventricular injection of GALP produces a dichotomous action that involves transient hyperphagia followed by hypophagia and a reduction in body weight^8^, whereas, in mice, it has only one action that reduces both food intake and body weight^9^. Intracerebroventricular GALP also causes a dose-related increase in core body temperature in rats and mice, suggesting an increase in sympathetic nervous system activation^8^,^10^. These results indicate that GALP replacement therapy could represent an effective treatment for obesity. Recently, it has been reported that intranasal leptin reduces food intake and body weight in diet-induced obese animals^11^. This demonstrates that it is feasible to convey large molecules such as leptin (16 kDa) to the brain via a less invasive approach than intracerebroventricular injection. Using radioactively iodinated GALP it has also been shown that, in comparison with intravenous treatment, intranasal administration provides a 40- to 100-fold improvement in targeting the brain versus peripheral tissues^12^. The first goal of this study was to investigate the targets of GALP in the brain when rats were intranasally administered iodinated GALP. The second aim was to clarify the effect of intranasal GALP on feeding behavior in lean and obese mice.

## Results

### Autoradiography

**Figure 1:**
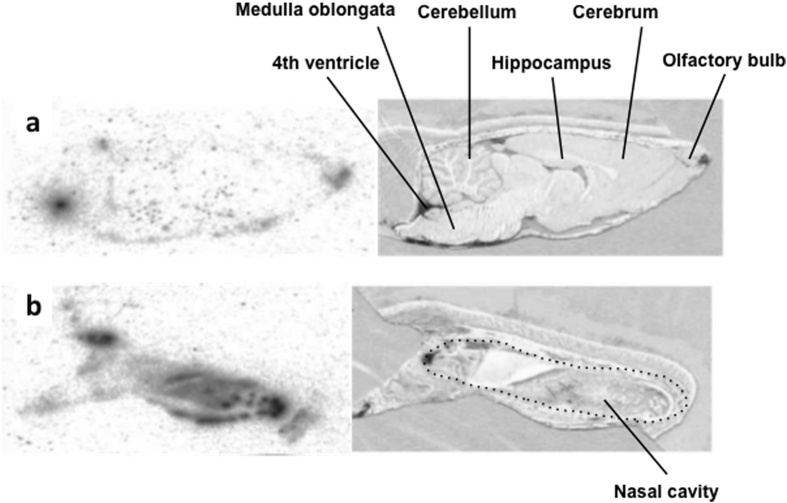
Autoradiographs of sagittal sections of brains 5 min after ^125^I-GALP administration. (**a**) Autoradiography of the brain +1.40 mm lateral to the median plane (left) and light microscopic image of the same section (right). (**b**) Autoradiography of the nasal region +1.40 mm lateral to the median plane (left), and light microscopic image of the same section (right).

Following intranasal administration,^125^I-GALP was observed as black spots dispersed throughout the brain, including in the olfactory bulb and the 4th cerebral ventricles (Fig. 1a). However, it was not concentrated in specific regions (Fig. 1a). Uptake into the nasal cavity was also observed (Fig. 1b).

### Dose-response in anesthetized ICR mice

**Figure 2:**
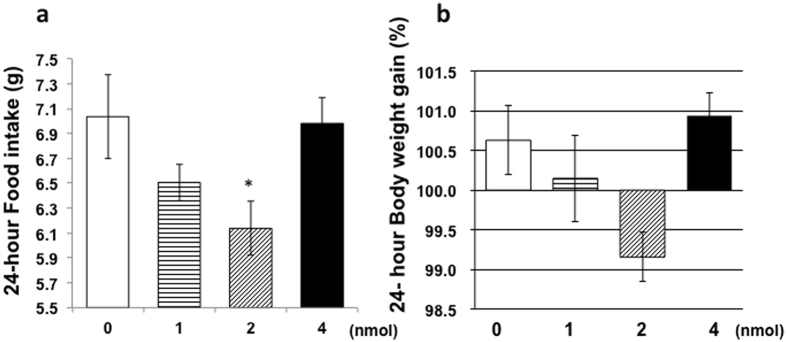
Change in food intake and body weight in response to intranasal administration of GALP. (**a**) Food intake and (**b**) body weight gain 24 h after the intranasal administration of vehicle (no fill, n = 3) or GALP (1 (horizontal lines), 2 (hash pattern) or 4 nmol (filled), n = 4 per group). The data are expressed as mean ± s.e.m., and analyzed by applying an ANOVA followed by Dunnett’s test, with comparison versus the vehicle group. *p < 0.05 vs. vehicle-treated mice.

To determine the effect of intranasal GALP treatment, mice were intranasally administered (1900 h) either vehicle (n = 4) or 1 nmol (n = 4), 2 nmol (n = 4), or 4 nmol GALP (n = 4) under anesthesia. Body weight, food intake, and water intake were measured at 24 h, and locomotor activity was monitored for 24 h after the treatment. Only the 2 nmol GALP-treated group displayed a significant decrease in food intake in comparison with the vehicle-treated group (Fig. 2a), with a concomitant trend towards decreased body weight (p = 0.07) (Fig. 2b). The food intake of the unanesthetized mice was 7.28 ± 0.08 g before the first experimental day. The food intake of the anesthetized mice (vehicle control group) was 7.03 ± 0.33 g. Therefore, the effect of anesthesia alone on feeding behavior is small.

### c-Fos expression in the hypothalamus and effect on food and water intake and locomotion

**Figure 3:**
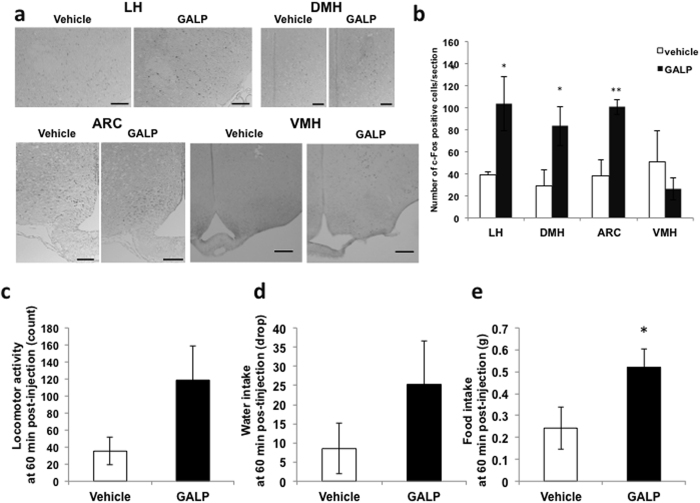
c-Fos expression in the hypothalamus 90 min post-injection, and food intake, water intake and locomotor activity. (**a**) Immunohistochemical staining of the hypothalamic region from vehicle- and GALP-treated mice. Scale bar in the lateral hypothalamus (LH) dorsomedial hypothalamus (DMH), and arcuate nucleus (ARC), 100 μm. Scale bar in the ventromedial hypothalamus (VMH), 200 μm. (**b**) Semi-quantitative scoring of c-Fos staining in the lateral hypothalamus (LH), dorsomedial hypothalamus (DMH), arcuate nucleus (ARC) and ventromedial hypothalamus (VMH) 90 min after intranasal administration of vehicle or GALP. The data are expressed as mean ± s.e.m., and analyzed by Student’s *t*-test. *p < 0.05 vs. vehicle treatment; **p < 0.005 vs. vehicle treatment. Locomotor activity (**c**), water intake (**d**) and food intake (**e**) 60 min after intranasal administration of vehicle (n = 4) or GALP (n = 4). The data are expressed as mean ± s.e.m., and analyzed by Student’s *t*-test was used. *p < 0.05 vs. vehicle-treated mice.

Compared to control vehicle administration, the intranasal administration of 2 nmol GALP led to a significant increase in c-Fos immunoreactivity in the lateral hypothalamus, dorsomedial hypothalamus and arcuate nucleus (Fig. 3a,b). However, no significant difference was observed in the ventromedial hypothalamus (Fig. 3b). There was no significant difference in locomotor activity or water intake between the vehicle- and GALP-injected groups in the first 60 min after treatment (Fig. 3c,d). Food intake in the GALP-treated group was significantly increased compared to that in the vehicle-treated group 60 min after injection (Fig. 3e).

### 7-day GALP treatment in *ob/ob* mice

**Figure 4:**
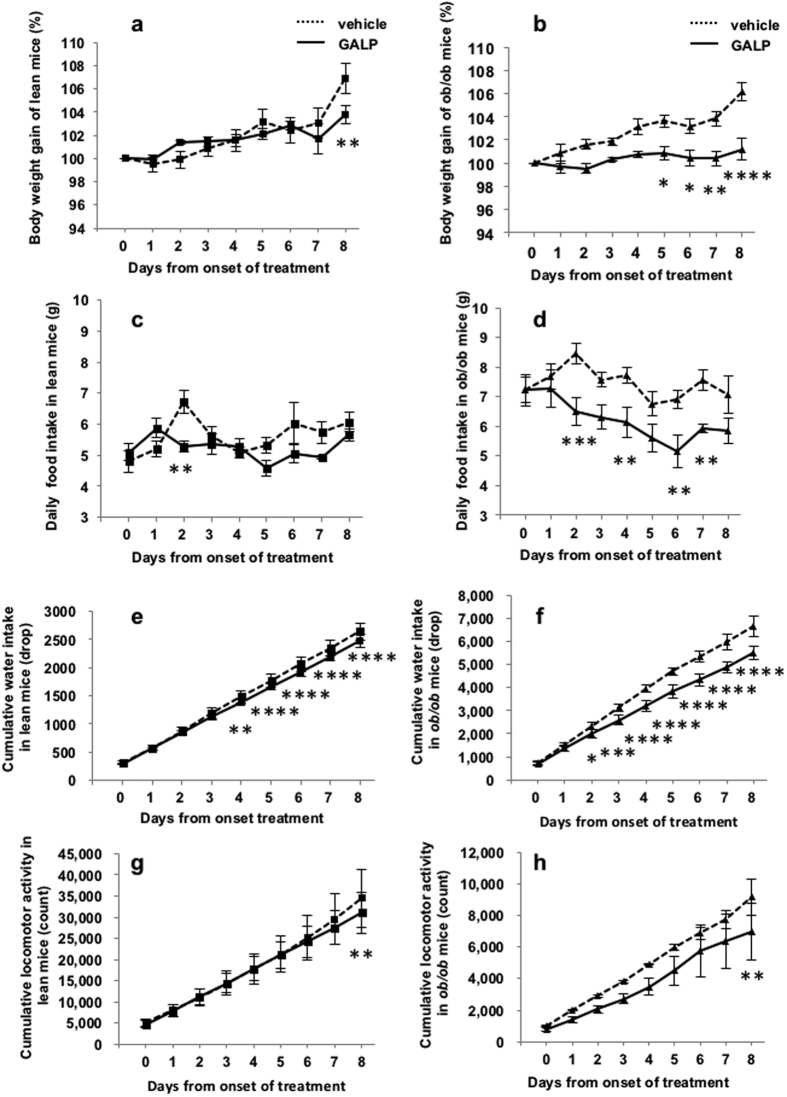
Effect of repeated intranasal GALP treatment in young *ob/ob* mice. (**a,c,e**,**g**) Show results from lean mice. (**b,d,f,h**) Show results from *ob/ob* mice. The effects of repeated intranasal administration of GALP on body weight gain (**a,b**), daily food intake (**c,d**), cumulative water intake (**e,f**) and cumulative locomotor activity (**g,h**) in lean (n = 4) and *ob/ob* (n = 4) mice at 13 weeks of age. The first GALP injection occurred on day 0. The data are expressed as mean ± s.e.m., and were analyzed by repeated two-way ANOVA followed by Bonferroni’s test. *p < 0.05 vs. vehicle-treated group; **p < 0.01 vs. vehicle-treated group; **p < 0.001 vs. vehicle-treated group; ****p < 0.0001 vs. vehicle-treated group.

Table 1 shows the body weight gain, food intake, water intake and locomotor activity in 13-week-old male C57BL/6 +/+ and *ob/ob* mice in the 24 h before the experiment. Body weight, food intake and water intake were significantly higher in the *ob/ob* mice than in the C57BL/6 +/+ mice, whereas the locomotor activity of the former was significantly lower. Blood glucose level were 3.5-fold higher in the *ob/ob* mice compared with wild-type animals. The C57BL/6 +/+ and *ob/ob* mice were then administered repeated intranasal doses of vehicle once a day for 7 days followed by 2 nmol of GALP once a day for 7 days while conscious. Body weight, food intake, water intake and locomotor activity were recorded 48 h after the final intranasal administration (i.e., day 8). Body weight gain in the lean mice did not differ significantly between the GALP and vehicle treatment periods (Fig. 4a). However, there was significant difference between the two groups 48 h after the final injection (Fig. 4a). In contrast, the body weights of obese mice gradually increased over the seven days of vehicle treatment, whereas subsequent administration of GALP significantly suppressed this spontaneous weight gain (Fig. 4b), an effect that lasted for 48 h after the final injection. The food intake of the GALP-treated lean mice was reduced compared with that of vehicle-treated mice 2 days after the onset of treatment (Fig. 4c), although this effect did not appear to persist. In contrast, the cumulative food intake in obese mice was significantly reduced by day 2 from the onset of treatment. Although there was no significant difference between the two groups 48 h after the final injection (Fig. 4d), food intake fell to the same level as that observed in lean mice 6 days after the onset of GALP administration (Fig. 4d). The water intake of obese was reduced throughout and beyond the treatment period, whereas that in lean mice was significantly reduced after 4 days (Fig. 4e,f). Locomotor activity in the both lean and obese mice did not differ significantly between the GALP and vehicle treatment periods (Fig. 4g,h). However, there was significant difference between the two groups 48 h after the final injection (Fig. 4g,h). Intranasally administered GALP did not affect blood glucose concentrations (Fig. 1S).

### 14-day GALP treatment in *ob/ob* mice

**Figure 5:**
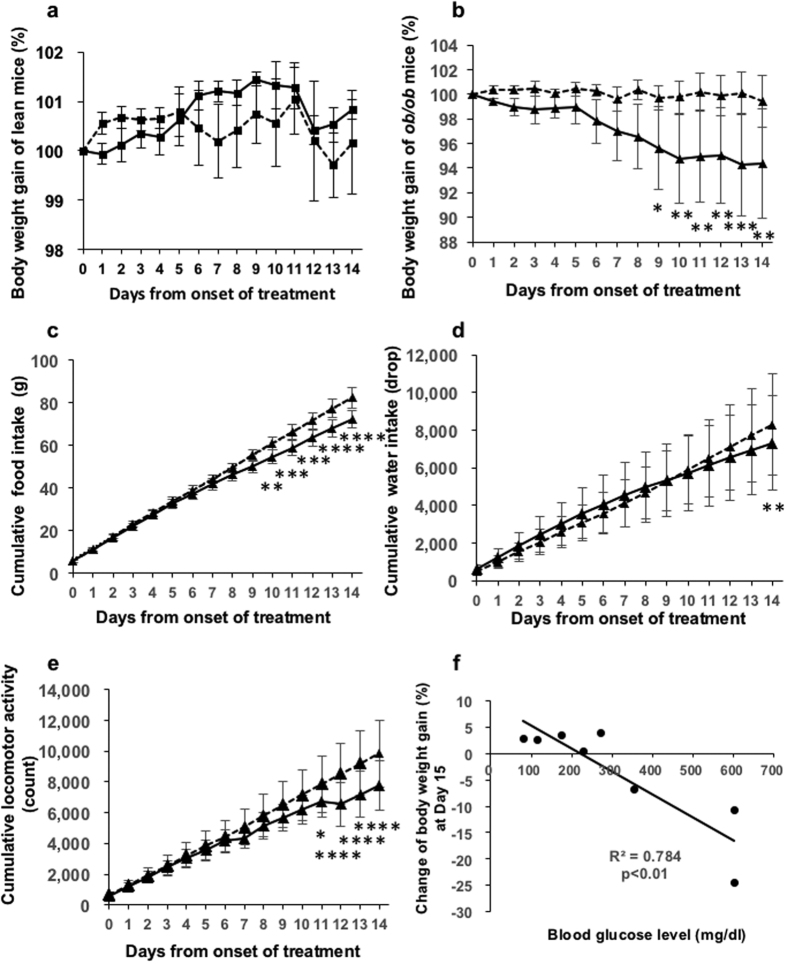
Effect of repeated intranasal GALP treatment in old *ob/ob* mice. Effects of repeated intranasal administration of GALP on body weight gain, daily food intake, cumulative water intake and cumulative locomotor activity in lean and *ob/ob* mice at 20 weeks of age. (**a**) Body weight gain in lean mice (n = 4). (**b**) Body weight gain, (**c**) cumulative food intake, (**d**) cumulative water intake, and (**e**) cumulative locomotor activity in *ob/ob* mice with hyperglycemia (n = 6). The first injection of GALP occurred on day 0. (**f**) Relationship between body weight gain 24 h after the 14^th^ intranasal administration of GALP and blood glucose level in *ob/ob* mice with hyperglycemia or normoglycemia (n = 8). The data are expressed as mean ± s.e.m., and analyzed by repeated two-way ANOVA followed by Bonfferoni’s test. The correlation analysis was performed using the Pearson’s product moment correlation coefficient. *p < 0.05 vs. vehicle-treated group; **p < 0.01 vs. vehicle-treated group; **p < 0.001 vs. vehicle-treated group; ****p < 0.0001 vs. vehicle-treated group.

Twenty-week-old male C57BL/6 +/+ and *ob/ob* mice were given repeated intranasal administrations of vehicle once a day for 14 days followed by 2 nmol of GALP once a day for 14 days. Body weight, food intake, water intake and locomotor activity were measured for 14 days from the onset of administration. There was no significant difference in body weight gain between the vehicle and GALP treatment periods in lean mice (Fig. 5a), nor were any significant differences seen with respect to food intake or locomotor activity (Fig. 2Sa). In obese mice, body weight gain in the GALP-treated mice was significantly reduced after 9 days (Fig. 5b). Significant differences were seen in cumulative food intake between the vehicle and GALP-treated obese group after10 days (Fig. 5c). However, cumulative water intake of the GALP-treated obese mice was significantly reduced on day 14 (Fig. 5d). Cumulative locomotor activity in the GALP-treated obese mice was significantly reduced by 11 days from the onset of treatment (Fig. 5e). Repeated intranasal administration of GALP did not affect blood glucose levels in *ob/ob* mice (Fig. 2Sb). As depicted in Fig. 5f, the plasma glucose concentration before the first injection was highly correlated with body weight gain at the end point in GALP-treated mice (r^2^ = 0.784, p < 0.01).

### 7-day GALP treatment of diet-induced obese mice

**Figure 6:**
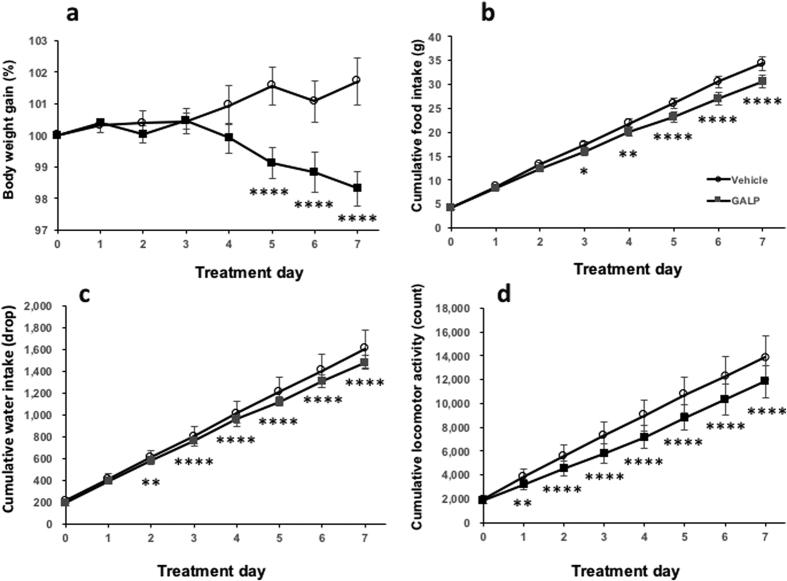
Effect of repeated intranasal GALP treatment in DIO mice. (**a**) Body weight gain, (**b**) cumulative food intake, (**c**) cumulative water intake, and (**d**) cumulative locomotor activity during 7 days of intranasal vehicle or GALP administration (n = 7 in both cases). The first injection of GALP occurred on day 0. The data are expressed as mean ± s.e.m., and were analyzed repeated two-way ANOVA followed by Bonferroni’s test. *p < 0.05 vs. vehicle-treated group; **p < 0.01 vs. vehicle-treated group; **p < 0.001 vs. vehicle-treated group; ****p < 0.0001 vs. vehicle-treated group.

Body weights of the diet-induced obese (DIO) mice were 52.4 ± 1.4 g on the first experimental day. The mice were intranasally administered vehicle once a day for 7 days, followed by 2 nmol GALP once a day for 7 days. This treatment led to a significant decrease in body weight by day 5 (Fig. 6a) and food intake by day 3 (Fig. 6b). Water intake was significantly reduced in the GALP-treated group by day 2 (Fig. 6d). Locomotor activity was significantly reduced throughout the GALP treatment period. (Fig. 6d). Furthermore, no significant difference in the plasma glucose level between the pretreatment and the 7^th^ GALP treatment was observed (140 ± 4 vs.159 ± 6 mg/dl, respectively, p = 0.19) (Fig. 3Sa). The plasma glucose concentration before the first dose of GALP correlated with the subsequent body weight gain in the GALP-treated mice (r^2^ = 0.60, p < 0.05) (Fig. 3Sb).

### Conditioned taste aversion test

**Figure 7:**
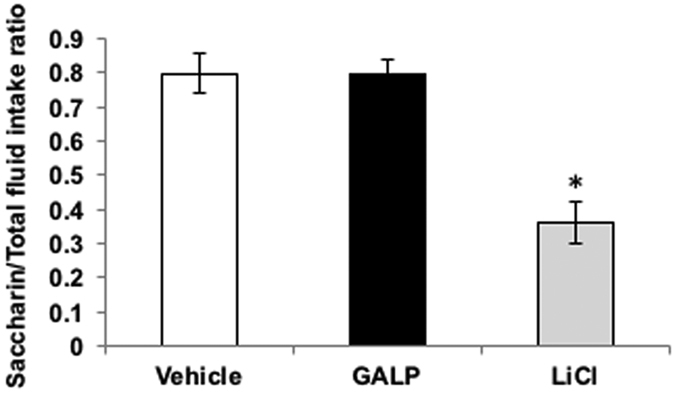
Conditioned taste aversion and food intake. Two-hour saccharin preference ratio (saccharin intake/total fluid intake (saccharin intake + water intake) %) in mice treated 48 h earlier with vehicle (intranasal administration, n = 4 (no fill)), GALP (2 nmol, intranasal administration, n = 4 (black fill)) or LiCl (0.15 M, intraperitoneal injection, n = 4 (grey fill)). The data are expressed as mean ± s.e.m. Differences between groups were assessed by one-way ANOVA, followed by Bonferroni’s test for multiple comparisons. *p < 0.05 vs. vehicle-treated mice.

Intraperitoneal injection of LiCl induced the formation of a conditioned taste aversion to saccharin, with LiCl-treated mice displaying a low saccharin/fluid intake ratio (p < 0.05, vs vehicle-injected group) (Fig. 7). In contrast, the intranasal administration of vehicle or GALP had no such effect (Fig. 7).

## Discussion

In this study, we found that intranasally injected GALP was transported from the nasal cavity to the microcirculation in the brain. Repeated administration of GALP decreased food intake, water intake, body weight gain and locomotor activity in *ob/ob* mice without taste aversion. A similar effect (decreased body weight gain, food intake and locomotor activity) was also observed in DIO mice in response to GALP treatment for 7 days.

Many peptides administered into the brain are then absorbed into the bloodstream and reach organs in the periphery^13^,^14^. Several physiological actions occur via crosstalk between central and peripheral peptides. In this study, the amount of GALP absorbed into the circulation was not measured because a suitable method has not been established. Nevertheless, the patterns of distribution of radiolabelled GALP in the cervical lymph nodes and spleen of mice are similar for intranasal and intracerebroventricular administrations^12^, suggesting that the same pathways are followed by GALP to enter the circulation. However, the levels of labelled GALP in the cervical lymph nodes and spleen is much lower following intranasal compared with intracerebroventricular administration, indicating that much less GALP effluxes from the brain to the blood in the former case^12^. Therefore, the contribution of peripheral GALP to the observed effects seen here is small at best.

Compared to intravenous administration, the intranasal administration of GALP has been shown to increase uptake into the brain^12^. The levels of uptake in the hypothalamus were about 10 times higher in the latter case. Cyclodextrins are cyclic oligosaccharides that consist of (α–1,4)-linked α-D-glucopyranose units. Cyclodextrin is frequently used as a carrier molecule to increase the solubility and absorption of small molecules such as peptides. Cyclodextrins have three different cavity sizes that consist of six, seven, or eight glucopyranose units, forming α-, β- and γ- cyclodextrin, respectively. Combining intranasally administered GALP with α-cyclodextrin increased uptake in the olfactory bulb and hypothalamus approximately 3- and 1.5-fold, respectively^12^. Therefore, α-cyclodextrin was added to the GALP solution in order to efficiently deliver GALP into the hypothalamus.

To demonstrate whether or not intranasally administered GALP activates neurons in the hypothalamus, we assessed c-Fos expression in this region, and found enhanced c-Fos expression in the nuclei involved in the promotion of feeding behavior, such as the dorsomedial and lateral hypothalamus and the arcuate nucleus. We also found that food intake increased 1 h after intranasal administration of GALP. On the other hand, intracerebroventricularly administered GALP has been shown to induce c-Fos expression in the parenchyma surrounding the ventricles, the ventricular ependymal cells and the meninges but not in the supraoptic nucleus, dorsomedial hypothalamus, lateral hypothalamus or nucleus tractus solitarius in mice^3^. The intracerebroventricular injection of GALP into mice has been reported to have no effect on feeding over 1 h 3 or to result in decreased food intake at 1 h 2, with neither centrally nor intranasally administered GALP affecting food intake 2 h post-administration^2^. Differences in results from intracerebroventricular and intranasal administration at 60 min may be due to differences in the transport of GALP into the cerebrospinal fluid or hypothalamus via the brain microcirculation. Taken together, these findings show that GALP is delivered to the feeding-related nuclei of the hypothalamus following intranasal administration, but that these nuclei are transient targets of GALP.

Although intracerebroventricularly administered GALP transiently increases food intake in rats, food intake and body weight were decreased 24 h after injection^9^. This transient hyperphagia can be explained by the fact that GALP activates orexin neurons in the lateral hypothalamus and neuropeptide Y neurons in the dorsomedial hypothalamus^15^,^16^. Both types of neuron contain orexigenic peptide. In the present study, intranasally administered GALP induced c-Fos expression in the lateral hypothalamus, dorsomedial hypothalamus and arcuate nucleus of the hypothalamus. However, in mouse, intracerebroventricularly administered GALP does not induce c-Fos expression in neurons in the lateral hypothalamus or dorsomedial hypothalamus. Therefore, it is possible that intranasally administered GALP activates orexigenic neurons in the lateral hypothalamus and dorsomedial hypothalamus in mice. On the other hand, GALP-induced hypophagia can be explained by the fact that intracerebroventricularly injected GALP has no significant effect on food intake or body weight in interleukin-1 receptor I knockout mice at 24 h compared with vehicle-administered^17^. These results indicate that interleukin-1 mediates the anorectic action of GALP via interleukin-1 receptor I in mice. Therefore, it is possible that intranasally administered GALP also reduces feeding behavior via the interleukin-1 signaling pathways.

In accordance with the study by Banks and colleagues^12^, ICR mice were used here to determine the dose response for GALP. Intranasally administered GALP at a dose of 1–2 nmol decreased food intake, whereas 4 nmol GALP had no such effect. Similar bell-shaped dose responses have been observed in the case of pituitary adenylate cyclase-activating polypeptide and neuropeptide Y^18^,^19^. These results suggest that the higher dose of GALP may down-regulate the expression of a specific receptor or decrease sensitivity to the peptide. Intranasally administered GALP most potently suppressed food intake at the 2 nmol dose, with a trend towards decreased body weight gain.

These findings determined the optimal dose for intranasal administration of GALP, and confirmed that centrally as well as intranasally administered GALP has an anorexigenic effect. Many studies have demonstrated that intracerebroventricular GALP has a suppressive effect on feeding behavior and a stimulatory effect on energy metabolism^8^,^9^,^20^. The repeated intracerebroventricular injection of GALP (twice a day for 4.5 days) transiently induces reductions in both food intake and body weight in wild-type mice on the first day, although these effects do not persist, suggesting that the animals become insensitive to repeated exposure to GALP^9^,^21^. However, in the present study, intranasally administered GALP had little effect on food intake, body weight gain or locomotor activity in young lean mice, highlighting the possibility of strain differences.

In the *ob/ob* mouse, long-term repeated GALP administration results in a sustained reduction in body weight, in spite of a significant recovery of food intake^20^. In young *ob/ob* mice, repeated intranasal administration of GALP at a lower dose (2 nmol) continued to reduce food intake, water intake and locomotor activity, and to suppress the expected spontaneous increase in body weight in comparison with repeated intracerebroventricular injection (5 nmol)^9^. This result suggests that repeated intranasal administration maintains GALP sensitivity. Locomotor activity is naturally lower in *ob/ob* mice than in lean mice. In spite of a further continuous decrease in locomotor activity seen here in response to GALP, the reduction in body weight was maintained. These findings suggest that not only a reduction in food intake but also the promotion of ongoing energy expenditure under leptin-deficient conditions contribute to the decrease in body weight.

The body weight of *ob/ob* mice reaches a plateau at 19 weeks of age. We found that intranasally administered GALP suppressed the increase in spontaneous body weight gain in young *ob/ob* mice. Intranasally administered GALP decreased body weight reached a plateau in aged *ob/ob* mice. Hyperglycemia in *ob/ob* mice gradually improves after 20 weeks of age^22^,^23^. When we divided the *ob/ob* mice into two groups (normoglycemic and hyperglycemic) on the basis of their blood glucose level on the first experimental day, we observed a subsequent negative relationship between blood glucose level and body weight gain. Our findings showed that intranasally administered GALP has a more pronounced effect on body weight loss in hyperglycemic compared to normoglycemic *ob/ob* mice. Thus, the decrease in body weight produced by intranasally administered GALP may be regulated by the plasma level of glucose, although GALP treatment did not affect this level. The explanation of the relationship between blood glucose level before the first injection and body weight gain therefore remains unclear.

Intranasally administered GALP also decreased food intake, water intake, body weight and locomotor activity in DIO mice, another model of obesity. Similar to the results obtained in aged *ob/ob* mice, the plasma glucose concentration before the first GALP injection correlated with body weight gain. These results suggest that the intranasal administration of GALP more effectively reduces body weight in obese mice with a higher initial blood glucose level. Intranasally administered GALP resulted in a decreased cumulative body weight in DIO mice in spite of a decreased in locomotor activity. The respiratory quotient produced by centrally administered GALP has been reported to be 0.7, meaning that GALP metabolizes lipid^24^,^25^. Intracerebroventricularly administered GALP increased expression of hepatic fatty acid βoxidation-related genes (carnitine palmitoyltransferase -1, medium-chain acyl-CoA dehydrogenase and acyl-CoA oxidase (AOX)) in the liver of C57BL/6 mice^25^. Therefore, these results suggest that intranasally administered GALP simultaneously suppresses energy intake and promotes energy expenditure without loss of sensitivity due to repeated exposure to GALP. In spite of the decrease in body weight produced by GALP, plasma levels remained unchanged in obese mice. Although centrally administered GALP has been shown to increase the gene expression of glucose transporter (GLUT)-4 which mediates glucose uptake in the gastrocnemius skeletal muscle, the blood glucose level was unchanged^24^. Centrally administered GALP also produces a trend towards increased gene expression of phosphoenolpyruvate carboxykinase that mediates the metabolic pathway of gluconeogenesis in the liver^24^. It is therefore possible that intranasally administered GALP aggravates insulin resistance, or that it promotes gluconeogenesis in the liver.

Elicitation of a conditioned taste aversion is widely used to assess whether or not a bioactive substance induces visceral discomfort in rats and mice^26^,^27^,^28^, although this effect remains a matter of debate^29^,^30^,^31^. To determine whether the anorectic effect of GALP was accompanied by visceral discomfort, a conditioned taste aversion paradigm was performed in obese mice. Similar to an intraperitoneal injection of LiCl, the intracerebroventricular injection of GALP has been shown to elicit the formation of conditioned taste aversion^9^. However, in our study, intranasally administered GALP did not elicit a taste aversion, suggesting that the observed decrease in food intake in obese mice was not due to visceral discomfort.

In summary, our novel findings are that GALP reduces body weight and food intake not only in *ob/ob* mice but also in DIO mice in response to repeated intranasal treatment, in spite of a decrease in locomotor activity. GALP also effectively reduces the body weight of obese mice with higher blood glucose levels. These results suggest that intranasal administration is an effective route whereby GALP can exert its effects as an anti-obesity drug.

## Materials and Methods

### Animals

Sprague-Dawley (SD) rats were purchased from Charles River Laboratories Japan, Inc. (Yokohama, Japan). ICR mice were purchased from Sankyo Labo Service Corporation, Inc (Tokyo, Japan). Both C57BL/6JHamSlc-*ob*/*ob* and C57BL/6JHamSlc-+/+ mice were purchased from Japan SLC, Inc (Hamamatsu, Japan). Diet-induced obese mice (C57BL/6J) were provided by Takeda Pharmaceutical Company (Osaka, Japan).

The animals were maintained on a 12:12 h light:dark cycle in a temperature-controlled room (lights on at 0800 h), with water and powder chow provided *ad libitum*. All experimental protocols were reviewed and approved by the Institutional Animal Care and Use Committee of Showa University. And all experiments were conducted according to the Guidelines for Animal Experimentation prepared by the Animal Care and Use Committee of Showa University.

### Autoradiography

Adult male SD rats were given a pre-surgical treatment to prevent them from swallowing intranasally administered GALP. The trachea was cannulated and connected to a ventilator under general anesthesia. Next, the esophagus was exposed and occluded with a surgical ligature. GALP (50 nmol/50 μl, Bachem AG, Bubendorf, Swizerland) was labeled with [^125^I]-Bolton-Hunter Reagent (100 μl/9.25 MBq) (Perkin Elmer, Waltham, MA). The animals were given a single intranasal administration of ^125^I-GALP (370 Bq/5.72 nmol/head) with 5% alpha-cyclodextrin, and euthanized with halothane 5 min later. The brains were removed and frozen in liquid nitrogen. Cryosections were prepared from each brain using a cryostat (CRYOMACROCUT, Leica Biosystems GmbH, Wetzlar, Germany). The intensity of ^125^I-GALP was measured using a bioimage analyzer (BAS-2000, Fuji Film, Tokyo, Japan).

### Intranasal administration of GALP into mice

Porcine GALP (1-60) (provided by Dr. Ohtaki of Takeda Pharmaceutical Company^1^ or purchased from Bachem AG) was dissolved in saline with 5% alpha-cyclodextrin, which inhibits GALP degradation^12^. A total of 2 μl was delivered to the cribriform plate by pushing a plastic 10 μl gel-loading pipette tip attached to a 10 μl micropipette through the right naris.

### Food and water intake, body weight gain and locomotor activity

Adult male mice were individually housed and habituated to the equipment used to measure feeding behavior (ACTIMO-100, Shinfactory Co., Ltd., Fukuoka, Japan) for approximately 1 week. The size of the living space was 240 mm wide, 290 mm long and 300 mm high. Food and water intake were recorded in each 1 min interval. Food intake was calculated according to the weight change of the chow box. Water intake was measured using a drop sensor. Sixty drops of water were approximately equal to 1 ml. Locomotor activity was monitored by 98 infrared beams at 20 mm intervals. Activity levels were expressed as the number of ambulations (two consecutive beam breaks) recorded in each 1 min interval. Body weight was measured at 24 h intervals.

### Determination of dose response using ICR mice

Adult male ICR mice were individually housed and habituated as above for approximately 1 week. To determine the dose response profile, the mice were anesthetized with 50 mg/kg of sodium pentobarbital (Dainippon Sumitomo Pharma Co., Ltd., Osaka, Japan) and received an intranasal administration (1900 h) of either vehicle (saline with 5% alpha-cyclodextrin, n = 3) or 1 nmol (n = 4), 2 nmol (n = 4), or 4 nmol GALP (n = 4). Food intake and body weight were measured 24 h after treatment.

### c-Fos expression in the hypothalamus

Adult male ICR mice were given a single intranasal administration of vehicle (saline with 5% alpha-cyclodextrin) or 2 nmol of GALP with alpha-cyclodextrin (1940 h). Ninety minutes after administration, the animals were anesthetized by an intraperitoneal injection of 50 mg/kg of sodium pentobarbital and transcardially perfused with saline, followed by a cold fixative solution containing 4% paraformaldehyde in 0.01 M PBS. The brains were removed, postfixed in the same fixative for 24 h at 4 °C, and transferred to a 0.01 M PBS solution containing 30% sucrose. The brains were frozen in isopentane-liquid nitrogen. Serial 7 μm-thick cryosections were prepared from each brain using a cryostat. Every fourth section was blocked with 5% normal goat serum for 1 h at room temperature (RT) and then incubated with rabbit anti-c-Fos antibody (1:30000, Ab-5; Oncogene Research Products, Cambridge, MA) followed by biotin-labeled goat anti-rabbit IgG antibody (1:200, Vector Laboratories, Inc., Burlingame, CA) for 2 h at RT. Nuclear c-Fos was visualized using the avidin-biotin-complex-3,3′-diaminobenzidine (ABC-DAB) method. To quantify the number of c-Fos-positive cells, sections were selected at 2.80 mm posterior to bregma. The number of c-Fos-positive cells in the visual field of the lateral hypothalamus, the dorsomedial hypothalamus, the arcuate nucleus and the ventromedial nucleus was counted.

### Feeding and water intake, body weight gain and locomotor activity in *ob/ob* mice

For 7 or 14 day treatment, 12- or 19-week-old male C57BL/6JHamScl- +/+ or C57BL/6JHamSlc-*ob/ob* mice were used, respectively. Adult male C57BL/6 +/+ and *ob/ob* mice were individually housed and habituated for approximately 1 week. Animals were given repeated intranasal administration of vehicle (saline with 5% alpha-cyclodextrin) once a day for 7 or 14 days followed by 2 nmol of GALP with alpha-cyclodextrin once a day for 7 or 14 days. Conscious mice were administered GALP at 1900 h (an hour before lights-out). Food and water intake and locomotor activity were recorded during the experiment. Body weight was measured every 24 h after intranasal administration.

### Feeding and water intake, body weight gain and locomotor activity in diet-induced obese (DIO) mice

Adult male C57BL/6 mice were fed a 45% high fat diet (Research Diets, D12331, New Brunswick, NJ) for 18 weeks. These mice were then individually housed and habituated for approximately 1 week. At 22 weeks of age, conscious DIO animals were given repeated intranasal administration of vehicle (saline with 5% alpha-cyclodextrin) once a day for 7 days, followed by 2 nmol of GALP with alpha-cyclodextrin once a day for 7 days at 1900 h (an hour before lights-out). Food and water intake and locomotor activity were recorded in each 1 min interval.

### Conditioned taste aversion study

The general experimental conditions were essentially the same as those previously described^32^. Six *ob/ob* mice received 2 h (1700–1900 h) daily access to water for 10 days to ensure stable fluid intake during the 2 h period. During this training period, mice were always given 2 bottles of water, which were pre-weighed. On day 10, the mice were again given 2 h access to water followed immediately by GALP (2 nmol, 1^st^ intranasal administration, n = 2) or vehicle (saline with 5% alpha-cyclodextrin, 1^st^ intranasal administration, n = 2) because GALP significantly decreased food intake after the second intranasal administration. On day 11 (for conditioned taste aversion), mice were given 2 h access to a 0.1% saccharin solution (rather than water), followed immediately by GALP (2 nmol 2^nd^ intranasal administration), LiCl (0.15 M in a volume equivalent to 2% of body weight, intraperitoneal administration) which served as a positive control for the formation of a conditioned taste aversion, or their respective vehicles (2^nd^ intranasal administration). Mice had one rest day in which they received 2 h access to 2 bottles of water. On the following day, they were presented with a bottle of water and a bottle of saccharin solution, and the fluid intake was measured at 30 min, 1 and 2 h. The placement of the bottles was changed at every reading time point, to avoid any side preference. Animals were then permitted free access to chow diet and restricted access to water for 5 days to allow the GALP and LiCl to be metabolized. Mice given vehicle were injected with GALP, and mice given GALP were injected with vehicle. Animals given LiCl were given LiCl again. The experiment was then repeated. The preference ratio was calculated as the intake of the drug-paired saccharin solution over the total liquid intake.

#### Measurement of plasma glucose

Tail snip blood glucose levels were measured using a glucose self-measuring kit (GR-102, Terumo, Tokyo, Japan).

#### Statistical analysis

All data are expressed as a mean ± s.e.m. for each group. Differences between two groups were assessed by Student’s t-test. Significant differences between groups were assessed by repeated two-way ANOVA followed by Bonferroni’s test for comparisons on the same day. Statistical comparisons of temporal changes in dose-response were performed using Dunnett’s test as compared with the vehicle (saline with 5% alpha-cyclodextrin)-treated group. The correlation analysis was performed using the Pearson’s product moment correlation coefficient. Differences between groups were assessed by one-way ANOVA followed by Bonferroni’s test for multiple comparisons in the conditioned taste aversion study. Statistical significance was defined as p < 0.05. All analyses were performed using GraphPad Prism version 7 (GraphPad Software, La Jolla, CA, USA).

## Additional Information

**How to cite this article**: Kageyama, H. *et al*. Anti-obesity effect of intranasal administration of galanin-like peptide (GALP) in obese mice. *Sci. Rep.* **6**, 28200; doi: 10.1038/srep28200 (2016).

## Supplementary Material

Supplementary Information

## Figures and Tables

**Table 1:** Initial profile in lean and *ob/ob* mice 24-h before the first experimental day.

	n	Body weight (g)	Food intake (g)	Water intake (drop)	Locomotor activity (count)	Blood glucose (mg/dl)
lean mice	4	22.4 ± 0.5	4.81 ± 0.35	311.3 ± 29.9	5186.5 ± 851.6	85 ± 7
*ob/ob* mice	4	38.9 ± 1.9***	7.22 ± 0.42***	742.3 ± 68.9***	1020.2 ± 40.3***	328 ± 35***

***p < 0.005 vs lean mice.
