# Supplementary material for: Anti-obesity effect of intranasal administration of galanin-like peptide (GALP) in obese mice
Source: Sci Rep. 2016 Jun 21;6:28200. doi: 10.1038/srep28200 (PMC4914964; doi:10.1038/srep28200)
Supplement: Supplementary Information [file srep28200-s1.doc]

Supplementary Information

Title of Manuscript

Anti-obesity effect of intranasal administration of galanin-like peptide (GALP) in obese mice

Authors

Haruaki Kageyama, Kanako Shiba, Satoshi Hirako, Nobuhiro Wada, Satoru Yamanaka, Yukinori Nogi, Fumiko Takenoya, Naoko Nonaka, Tsutomu Hirano, Shuji Inoue and Seiji Shioda

This file contains Supplementary Figure 1-3

Contents

Supplementary figure 1 Change of blood glucose level in response to intranasally administered GALP.

Supplementary figure 2 Effect of intranasal GALP treatment in old lean mice.

Supplementary figure 3 Effect of intranasal GALP treatment on blood glucose in DIO mice.


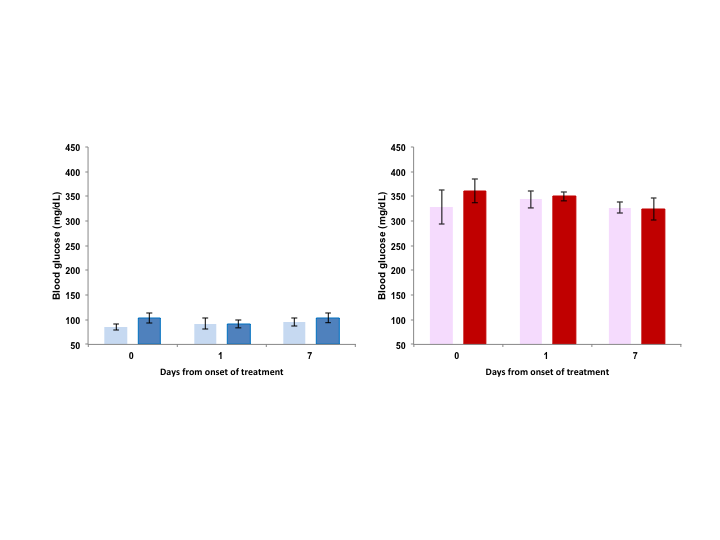


Supplementary Fig. 1 Change of blood glucose level in response to intranasally administered GALP.

Blood glucose was measured on the initial day, day 1 and day 7 (end point). Blue and red in a column indicate lean and *ob/ob* obese mice, respectively. Light and dark colors indicate the vehicle- and GALP-treated groups, respectively. The data were expressed as mean ± s.e.m., and analyzed by repeated two-way ANOVA followed by Bonfferoni’s test. No significant differences were observed.


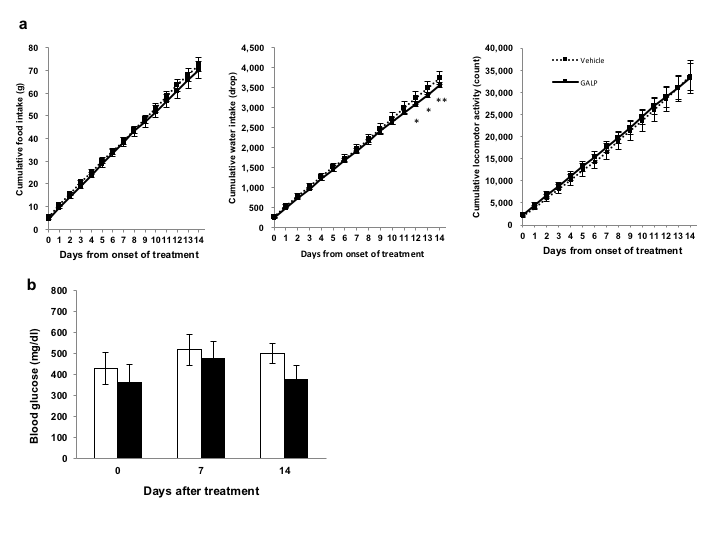
Supplementary Fig. 2 Effect of intranasal GALP treatment in old lean mice.

(a) Cumulative food intake, cumulative food intake and cumulative locomotor activity, and (b) blood glucose level in lean mice. Blood glucose was measured on the initial day, and days 1, 7 and 14 (end point). White and black columns indicate the vehicle- and GALP-treated groups, respectively (n = 4 per group). The data were expressed as mean ± s.e.m., and analyzed by repeated two-way ANOVA followed by Bonfferoni’s test. No significant differences in blood glucose were observed. * p<0.05 vs. vehicle-treated mice; ** p<0.01 vs. vehicle-treated mice.


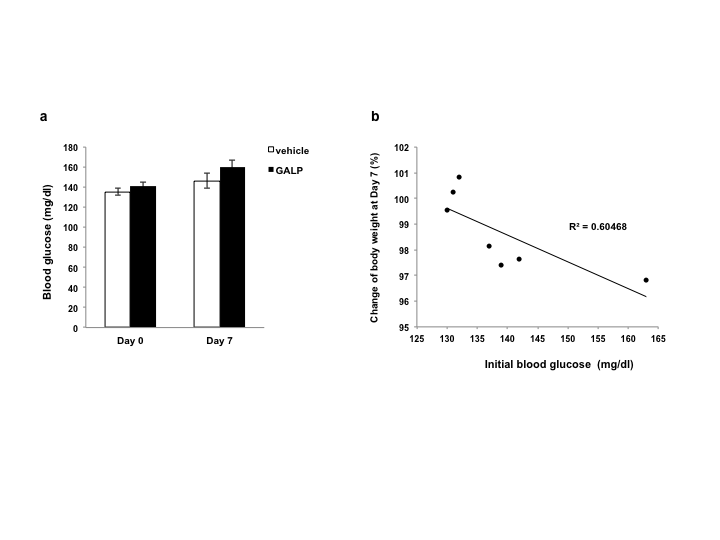


Supplementary Fig. 3 Effect of intranasal GALP treatment on blood glucose in DIO mice.

(a) Blood glucose concentration. White and black columns indicate the vehicle- and GALP-treated groups, respectively (n = 7 per group). The data were expressed as mean ± s.e.m., and analyzed by repeated two-way ANOVA followed by Bonfferoni’s test. No significant differences were observed. (b) Relationship between change in body weight 24 h after the 7^th^ intranasal administration of GALP and initial blood glucose level in DIO mice (n = 7). The correlation analysis was performed using the Pearson's product moment correlation coefficient. r^2^=0.60468, p<0.05
